# Kernel Method Based Human Model for Enhancing Interactive Evolutionary Optimization

**DOI:** 10.1155/2015/185860

**Published:** 2015-03-23

**Authors:** Yan Pei, Qiangfu Zhao, Yong Liu

**Affiliations:** The University of Aizu, Tsuruga, Ikki-machi, Aizuwakamatsu, Fukushima 965-8580, Japan

## Abstract

A fitness landscape presents the relationship between individual and its reproductive success in evolutionary computation (EC). However, discrete and approximate landscape in an original search space may not support enough and accurate information for EC search, especially in interactive EC (IEC). The fitness landscape of human subjective evaluation in IEC is very difficult and impossible to model, even with a hypothesis of what its definition might be. In this paper, we propose a method to establish a human model in projected high dimensional search space by kernel classification for enhancing IEC search. Because bivalent logic is a simplest perceptual paradigm, the human model is established by considering this paradigm principle. In feature space, we design a linear classifier as a human model to obtain user preference knowledge, which cannot be supported linearly in original discrete search space. The human model is established by this method for predicting potential perceptual knowledge of human. With the human model, we design an evolution control method to enhance IEC search. From experimental evaluation results with a pseudo-IEC user, our proposed model and method can enhance IEC search significantly.

## 1. Introduction

Interactive evolutionary computation (IEC) is an optimization method that can incorporate human knowledge into an optimization process. It converges to a solution accordingly with certain human preference. From a framework viewpoint, IEC can be implemented with any evolutionary computation (EC) algorithm by replacing the fitness function with a human user. General category of IEC methods includes interactive genetic algorithm (IGA) [1], interactive genetic programming [2], interactive evolution strategy [3], and human-based genetic algorithm [4]. There are many challenges in IEC researches and its applications. Reference [5] presented a review of research on IEC challenges. These research areas include discrete fitness value input method, prediction of fitness values, user interface for dynamic tasks, acceleration of IEC convergence, combination of IEC and non-IEC, active intervention, and IEC theoretical research. Utilization of IEC allows fusing human and computer for problem solving. However, taking the evaluation process into the hands of an user sets up a different scenario compared to normal optimization methods, and it leads to serious problems when putting IEC into practice. One of the problems is user fatigue in an evaluation process of the IEC.

It is necessary to relieve user fatigue for many IEC applications to improve performance of target systems. References [6, 7] presented to use semisupervised learning technique in IGA to enhance IEC search. Reference [8] embedded decision-maker's preferences in IEC in multiobjective optimization problem. Another solution to solve this problem is to accelerate IEC search by using fitness landscape directly [9]. Fourier transform is applied to obtain frequency information to analyze fitness landscape [10, 11]. A landscape approximation method with simpler shape was proposed, but the computational cost of approximation in an original high dimensional search space is costly [12]. Reference [13] presented an approximation method of projecting an original fitness landscape into each lower dimension. From comparison evaluation results, it can save computational cost significantly [14]. Dimensionality reduction method can obtain a fitness landscape in lower dimensional space to support useful information for search. This method has been applied to the travelling salesman problem in a real world application [15, 16]. On the other hand, if we project an original search space into a higher dimensional space by kernel method, we can also obtain useful information for finding optimum region. For an IEC application, kernel method is a tool to establish a human model.

When a human conducts an IEC experiment, fitness landscape of IEC is an approximate model of human evaluation landscape. It is different to establish an exact mathematical model to express IEC fitness landscape, that is, IEC user model, which is usually nonlinear, discrete, constraint, multimodal, noisy, and high dimension. The great difference between an IEC user model (the terms, “user model” and “human model,” have the same meaning in this paper. However, user model refers to a concept of individuality in physical level usually, and human model refers to a concept of abstraction in logic layer) and an ordinary fitness functions is in the implementations of (a) relative and (b) discrete fitness evaluations that are produced by a human user. Unlike an ordinary fitness function, a human IEC user compares given objectives in relative terms and never produces an absolute fitness value. He or she also cannot give precise fitness values, but rather can only rank according to discrete levels (e.g., 1 to 5 or 1 to 7 levels) every generation, while ordinary fitness functions give continuous values. When a difference between individuals is less than a minimum discrete fitness range, that is, an evaluation threshold, a human IEC user cannot distinguish the difference. Such difference becomes fitness noise that IEC user model should implement. Kernel method is a powerful tool that can project an IEC search space from its original discrete search space into a new higher dimensional space (*feature space*) by conducting a non-linear transformation with suitable kernel function. After then, we can use a linear model as a human model in the feature space to analyze human perceptual knowledge easily. The linear model in feature space corresponds to an original complex nonlinear model in an original IEC search space.

This paper proposes a method to obtain and analyze IEC human model in high dimensional search space by kernel classification method, which is beneficial to a discrete search space problem, such as IEC. First, we separate some individuals into two groups as training sample data. One group is near optimum with related better fitness, and the other group is beyond optimum with a worse fitness. Second, we project these individuals into high dimensional feature space by some kernel functions. Utilization of different kernel function is to map an original search space into different topological feature space. Third, in feature space, we establish a human model by a linear classifier to support correct classified fitness landscape that linear classifier in original search space cannot support. It is a novel method to establish a human model in IEC research and can be extended into IEC application in many perspective interdiscipline research. The method for establishing a human model presents an originality of this paper. With the obtained human model in a high dimensional search space, we propose an evolution control method to enhance IEC search and use four Gaussian mixture models as pseudo-IEC user to evaluate our proposed methods. From experimental evaluation results, our proposed evolution control methods can accelerate IEC search significantly.

The remainder of this paper is structured as follows.  Section 2 presents an overview of human model in computation.  Section 3 presents an overview of kernel method and introduces our linear classifier design method by kernel classification in detail. Some kernel functions used in our study are described, in which they are used to project an original search space to different feature space.  Section 4 proposes an evolution control method by using human model in feature space. Fitness landscape in feature space is studied and discussed. Evaluations are conducted in Section 5 by using four Gaussian mixture models as pseudo-IEC user to evaluate our proposed methods. Some discussions of our proposed method and evaluation results and several open topics are presented in Section 6.  Section 7 concludes the whole paper while some future work is presented.

## 2. Human Model in Computation

### 2.1. Human Related Computation

Some computation mechanisms need human assistance to complete a certain task, since the human's capability and preference were introduced into computational process. The key words that relate to these researches and applications are games, interactive optimization, and human computation interaction, and so forth. On the one hand, human has intelligence, such as non-linear thing, productivity, innovation, hypothesis, that the computer or computation cannot simulate and compute. Human can therefore compensate these drawbacks of computer or computation. On the other hand, the computer has powerful and huge computation capability. The computer can help a certain user to complete their works in computational way of releasing their workloads and fatigues. The crucial issue is type and way in designing these human and computation cooperations.

The prototype and mechanism related to human and computation can be categorized into three perspectives. Firstly, it uses human's intelligence, computational capabilities, and advantages of computer to compensate each sides' limitations. Human and computer work together for a certain task. It is the subjective of human computer cooperation. Secondly, it obtains human's potential or unknown knowledge (e.g., psychological, physiological, or intelligent knowledge) from a computational process. It belongs to the topics of humanized information extraction from interaction between human and computer. Thirdly, it enhances human cognitive competence by a computational process. It is intelligence amplification [17].

Human model is an essential research subject in researches of computational process related to human, such as human computation, awareness computing, and IEC. In human computation technique, two prominent human-computation techniques, games with a purpose and microtask crowd sourcing, can help resolve semantic technology related tasks, including knowledge representation, ontology alignment, and semantic annotation [18]. Human model can improve these computations and be applied to further commercial outlook. In awareness computing, human model can be introduced into its computational process to analyze human awareness mechanism [19]. In IEC, human model can be used either to analyze human cognitive knowledge in human side or to enhance IEC search in the computer side for releasing user fatigue. This is the subject of this paper as well.

**Figure 1:**
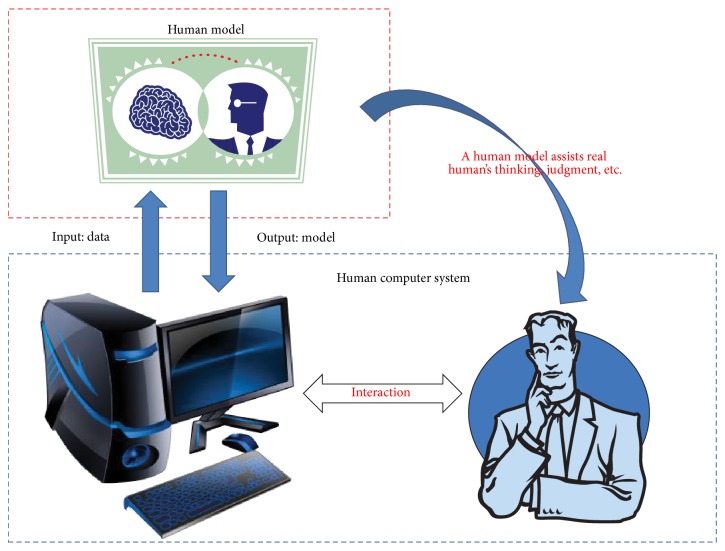
Conceptual diagram of a human model, which can assist a real human in a human computer system. From the interaction between human and computer, we can obtain the relationship of human's preference, thinking, knowledge, and so forth, and its related elements in computer system. And then, we use these data to establish a human model in the computer to assist human to solve a certain task or problem.

Figure 1 demonstrates a conceptual diagram of human model that can assist and extend the human's capability in a human computer system. There are two components in this diagram: one is a human model and the other is a human computer system. The human computer system supports learning data to a human model system, which can establish a human model to help a real human to complete a certain work in an environment of interaction between human and computer. The implementations of human model present its research philosophy.

### 2.2. Human Model

Model and simulation are important for theoretical study in human related computation. Any success of practical applications comes from fundamental research with the necessary assistance of model and simulation. When a system relates to a real human, it is essential to establish a human model to simulate characteristics of a human for research. Generally, the Turing machine can be considered as the first human model in the history of computer science [20]. There are three aspects in a human model, that is, perceptual model, cognitive model, and physical model [21]. They correspond to concepts of sensation and perception, consciousness, and behavior in psychological research.

We should clearly define psychological and physiological characteristics of each layer to establish a human model, which is as well as a research issue in ergonomics research. In every aspect of each layer, there are different research scales for a human model. In perceptual model, it can be separated into vision model, auditory model, olfactory model, and gustatory model. In cognitive model, it can be separated into space model, time model, motion model, emotion model, and so forth. A study on human emotion and cognition recognition was conducted by soft computing techniques [22]. In physical model, it can be separated by functions or organs, and so forth. A human organ model was established to better understand human physiological activities and disasters themselves [23].

Human model is a synthesis model of perceptual and cognitive model mentioned above, which is established for a certain computational tasks. It has a variety of implementations, whatever the purposes and the forms come from. Not only human but also animal has computational capability in the world [24, 25]. The study scale of human model can be extended into the life world as a life model to recognize and understand the behaviour of the natural world. Some of primary discussable issues in natural computing are related to this topic [26]. The prior issue of establishing a human model is to distinguish differences between pure computing and human thinking [27].

## 3. Kernel Methods

### 3.1. Kernel Trick

Kernel methods present a series of data transformation techniques in machine learning that projects original space data into another higher dimensional space, that is,* feature space*, in which we can establish a linear model to reduce complexity of data relation. Typically, kernel methods are applied in classification and regression problems [28].

In general, linearity is a special characteristic, and no model of a real system is actually linear. However, linear relations have been focused in many research areas. If a model is nonlinear, we can project it into a feature space for obtaining linear relation, but not trying to fit a nonlinear model in an original space [12]. This kind of techniques are known as kernel trick.

The kernel trick was originally proposed in [29]. Mercer's theorem is its mathematical result, which presents that any continuous, symmetric, positive semidefinite kernel function *K*(*x*, *y*) can be expressed as an inner product 〈*x*, *y*〉 form in a high dimensional space [30]. Suppose that there are sample data (1) in a measurable space *P*, the kernel is positive semidefinite (2). There must be a function *φ*(*x*), that is,* feature map*, whose range is in an inner product space *Y* of high dimension, shown in (3). This transformation process can be expressed in (4):

(1) $$\begin{array}{c} \text{SampleData}=x_{0},x_{1},\ldots ,x_{n}\in P, \end{array}$$

(2) $$\begin{array}{c} \underset{i,j}{\sum }K\left(x,y\right)c_{i}c_{j}\geq 0, \end{array}$$

(3) $$\begin{array}{c} K\left(x,y\right)=\langle x\cdot y\rangle , \end{array}$$

(4) $$\begin{array}{c} P⟶\varphi \left(x\right)⟶Y. \end{array}$$

There are several advantages of kernel methods. First, the kernel methods define a similarity measurement among sample data and present original space complex information in a simple form in feature space. Second, its computational complexity depends on the kernel function only and does not utilize feature map and feature space explicitly. Third, the kernel methods use training data in the form of kernel function and kernel matrix rather than the training data themselves, because there is no need to conduct a feature map explicitly in a high dimensional feature space.

### 3.2. Kernel Classification

Mercer theorem presents that kernel function corresponds to some feature space, and its mathematical result was presented in [30]. Since it is proposed, kernel methods were used in wide research areas, which include classification [31], principal component analysis [32], pattern analysis [33], support vector machine [34], and so forth.

**Figure 2:**
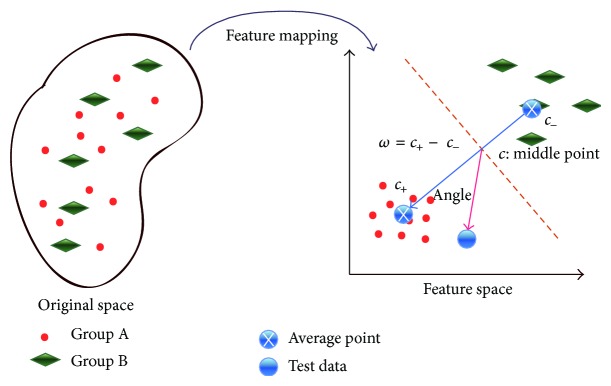
Human model by a binary classifier in our experiments. These two categories cannot be separated linearly in original space; however, after projecting these data into a higher dimensional feature space, they can be separated by a binary classifier. The linearity characteristic shows the advantage of proposed human model.

Kernel classification processes the data that is difficultly distinguished in an original space. It projects them into higher dimensional space by kernel function to design proper linear classifier for solving classification problems. In our proposal, we consider human model as a simple binary classification problem as in Figure 2. The human model is applied to IEC for modelling characteristics of an IEC human user and enhancing IEC optimization.

In Figure 2, two category data properties are hard to be separated into two groups by a linear model in an original space. By a feature map, the data in original space is transferred into a high dimensional feature space, where they can be separated into two groups by a linear model easily. The binary classification problem in the original space and feature space is described by (5) and (6), respectively,

(5) $$\begin{array}{c} \left\{\left(x_{1},y_{1}\right),\left(x_{2},y_{2}\right),\ldots ,\left(x_{n},y_{n}\right)\right\}\in R^{d}\times \left\{+1,-1\right\}, \end{array}$$

(6) $$\begin{array}{c} \left\{\left(\varphi \left(x_{1}\right),y_{1}\right),\left(\varphi \left(x_{2}\right),y_{2}\right),\ldots ,\left(\varphi \left(x_{n}\right),y_{n}\right)\right\}\in H\times \left\{+1,-1\right\}. \end{array}$$

In feature space, we can obtain a vector *ω*  (9), which is from one group vector's center vector (7) to the other (8), and its middle point is shown in (10). When new unknown data comes, we can judge its category through the angle of *ω* and the vector from *ω*'s middle point to the unknown point. The concrete judgement process is shown in (11), where sgn is a sign function. If the result is positive, the unknown data belongs to positive group; conversely, it belongs to the other group (negative group),

(7) $$\begin{array}{c} c_{+}=\frac{1}{m_{+}}\overset{m_{+}}{\underset{i=1}{\sum }}\varphi \left(x_{i}^{+}\right), \end{array}$$

(8) $$\begin{array}{c} c_{-}=\frac{1}{m_{-}}\overset{m_{-}}{\underset{j=1}{\sum }}\varphi \left(x_{j}^{-}\right), \end{array}$$

(9) $$\begin{array}{c} \omega =c_{+}-c_{-}, \end{array}$$

(10) $$\begin{array}{c} c=\frac{1}{2}\left(\omega \right)=\frac{1}{2}\left(c_{+}-c_{-}\right), \end{array}$$

(11) $$\begin{array}{c} y=\mathrm{sgn}\left(\langle \varphi \left(x\right)-c,\omega \rangle \right), \end{array}$$

(12) $$\begin{array}{c} =A-B-\frac{1}{2}\left(C-D\right). \end{array}$$

In feature space, we can judge the unknown data's category through (11). However, there is not an explicit feature map *φ* in kernel classification method, we must establish the feature map *φ* with some kernel function form. Equation (13) shows the concrete algorithm of (11) with the forms of kernel function,

(13) $$\begin{array}{c} A=\frac{1}{m_{+}}\overset{m_{+}}{\underset{i=1}{\sum }}K\left(x_{i}^{+},x\right),\\ B=\frac{1}{m_{-}}\overset{m_{-}}{\underset{j=1}{\sum }}K\left(x_{j}^{-},x\right),\\ C=\frac{1}{m_{+}^{2}}\overset{m_{+}}{\underset{i=1\;}{\sum }}\overset{m_{+}}{\underset{i=1}{\sum }}K\left(x_{i}^{+},x_{i}^{+}\right),\\ D=\frac{1}{m_{-}^{2}}\overset{m_{-}}{\underset{j=1\;}{\sum }}\overset{m_{-}}{\underset{j=1}{\sum }}K\left(x_{j}^{-},x_{j}^{-}\right). \end{array}$$

### 3.3. Kernel Functions

The selection of kernel function is a crucial issue for the success of all kernel algorithms, because the kernel function constitutes prior knowledge that is available about a task. Accordingly, there is no free lunch in kernel function selection. In our proposed human models and evolution control methods, we use three well known kernel functions with different parameters in our experimental evaluation. They are linear kernel, polynomial kernel and Gaussian kernel (radial basis function, i.e., RBF kernel). Equations (14), (15), and (16) show their concrete forms:

(14) $$\begin{array}{c} K\left(x,z\right)=\langle x,z\rangle , \end{array}$$

(15) $$\begin{array}{c} K\left(x,z\right)={\left(\langle x,z\rangle +1\right)}^{r}, \end{array}$$

(16) $$\begin{array}{c} K\left(x,z\right)=\exp\frac{-{\left|x-z\right|}^{2}}{2\sigma^{2}},\;\sigma \in R-\left\{0\right\}. \end{array}$$

## 4. Kernel Classification Based Human Model

### 4.1. Concept of the Proposal

In an original search space, we cannot basically utilize a linear classifier to judge an individual's property by fitness, which is preference of a certain user in IEC. That means it is impossible to establish a linear human model in original search space. Reference [9] reported a dynamic fitness threshold technique to ensure fitness increasing from one generation to the next. However, there is possibility to lead to local optima due to the fact that classifier model is linear in an original search space. Reference [35] proposed a constructive mapping genetic algorithm (CMGA) to implement this mechanism.

In Figure 2, suppose that circles show the better fitness area and rhombuses are the worse fitness area. If we use a dynamic fitness threshold technique (such as CMGA), which is linear in original search space, to filter new offspring, many individuals with better fitness will be drawn up so that algorithm performance will become worse. However, in feature space, this dynamic fitness threshold technique can be implemented by a linear classifier thanks to projecting them into a higher dimensional search space by kernel method. All individuals can be separated clearly and exactly in feature space, and this is beneficial to obtain a fitness landscape in high dimensional feature space. For IEC, it is a human model that is implemented by a linear classifier in feature space.

### 4.2. Evolution Control Method by a Human Model

Accordance with the primary motivation and kernel classification method, we design an evolution control method by establishing a human model in feature space to enhance IEC search for relieving user's fatigue. The proposed algorithm is shown in Algorithms 1 and 2.  Algorithm 1 is a framework of kernel method based GA, and Algorithm 2 is one of its implementations by using a human model.

#### 4.2.1. Training Data Selection

It is crucial to select labelled training data in an original search space to distinguish the individuals' category (i.e., human preference). We choose *n* and *m* different individuals with the better fitness and the worse fitness as training data for kernel function, which shows ones are near the optimum and the others are beyond the optimum. The parameters *n* = 3 and *m* = 3 are in experimental evaluation. The performance of selection method depends on kernel function design corresponding to a certain fitness landscape in feature space or a certain human user preference.

#### 4.2.2. Human Model Implementation by a Linear Classifier Based on Kernel Method

After we obtain the labelled training data as input data for kernel function, we project these labelled training data (individuals and their fitness) into high dimensional feature space by a kernel function. In our experimental evaluation, we use three kernel functions, which are shown in (14), (15), and (16). Parameter setting is that *r* = 2,3, 5,7 is in polynomial kernel, and *σ* = 1,3, 5,7 is in RBF kernel for comparing their optimization performance. The training and implementation of linear classifier are from one generation to the next. It is an online training and utilization method, which can adapt IEC user preference from one generation to the next.

The utilization of different kernel function is conducing an operation to map individuals into a different dimensional and structural feature space, so the performance of linear classifier (human model) design is decided by the selection of kernel function and its parameter setting. In Algorithm 1, Step (6) shows a linear classifier training in every generation. For a certain fitness landscape in an original search space, there must be an optimal linear classifier (human model) design with a certain kernel function and its parameter setting. It is a promising study topic to obtain this optimal human model for IEC search. We will conduct this research topic in the future.

#### 4.2.3. Human Model Utilization

In a high dimensional feature space, we establish a human model by a linear classifier to support preference of a human user that linear classifier in an original search space cannot support. When IEC obtains a new offspring, that is, an object for evaluation, we use the designed human model to classify its category (near or beyond global optimum, i.e., human subjective preference) and then to judge whether to put it into the next generation (Algorithm 2). Because this processing is conducted by computer automatically, it does not increase human evaluation workload, but can enhance IEC search significantly. The training and utilizing human model can be applied every generation or several generation once. In our experimental evaluation, we evaluate our proposal with the first method, that is, training and utilizing human model every generation.

## 5. Experimental Evaluation

### 5.1. Simulation Experimental Design

User fatigue is a considerable factor in the IEC optimization evaluation. Experimental evaluations frequently require many repeated experiments under the same conditions, and in this case it is necessary to perform the evaluations using an IEC user model rather than with a real human IEC user. We need to evaluate acceleration methods by analyzing the load of a single evaluation along with the convergence characteristics through IEC simulation. After that we must conduct a human subjective evaluation to evaluate user fatigue and acceleration performance synthetically and thus conclude our evaluation of methods proposed here. This paper deals with IEC simulation of the first stage.

Gaussian mixture model is modelled as a pseudo IEC user in our simulation in Section 5.2. We conducted simulation evaluations to compare the characteristics of several methods with multiple different initializations under the same experimental conditions. A constructive mapping genetic algorithm (CMGA) is introduced as a comparison algorithm in our experiment in Section 5.3. We explain our proposed algorithms, their parameter setting, and evaluation metrics, such as several statistical tests in Section 5.4. Some evaluation results and observations are initially summarized in Section 5.5,

(17) $$\begin{array}{c} \text{GMM}\left(x\right)=\overset{k}{\underset{i=1}{\sum }}a_{i}\exp\left(-\overset{n}{\underset{j=1}{\sum }}\frac{{\left(x_{ij}-\mu_{ij}\right)}^{2}}{2\sigma_{ij}^{2}}\right), \end{array}$$

(18) $$\begin{array}{c} \sigma =\left(\begin{array}{cccccccccc} 1.5 & 1.5 & 1.5 & 1.5 & 1.5 & 1.5 & 1.5 & 1.5 & 1.5 & 1.5\\ 2 & 2 & 2 & 2 & 2 & 2 & 2 & 2 & 2 & 2\\ 1 & 1 & 1 & 1 & 1 & 1 & 1 & 1 & 1 & 1\\ 2 & 2 & 2 & 2 & 2 & 2 & 2 & 2 & 2 & 2 \end{array}\right), \end{array}$$

(19) $$\begin{array}{c} \mu =\left(\begin{array}{cccccccccc} -1 & 1.5 & -2 & 2.5 & -1 & 1.5 & -2 & 2.5 & -1 & 1.5\\ 0 & -2 & 3 & 1 & 0 & -2 & 3 & 1 & 0 & -2\\ -2.5 & -2 & 1.5 & 3.5 & -2.5 & -2 & 1.5 & 3.5 & -2.5 & -2\\ -2 & 1 & -1 & 3 & -2 & 1 & -1 & 3 & -2 & 1 \end{array}\right), \end{array}$$

(20) $$\begin{array}{c} a_{i}={\left(3.1,3.4,4.1,3\right)}^{T}. \end{array}$$

### 5.2. Gaussian Mixture Model as a Pseudo-IEC User

Reference [36] discussed some limits of human brain with respect to information processing. In particular, this research had found that people are unable to keep up with more than 5–9 different chunks of information at one time. Gaussian mixture model (GMM) with less dimensional setting can well simulate it and some features of evaluation when a human conducts an IEC experiment, that is, relative and discrete fitness evaluations. GMM consists of different mean, variance, and peak together to express the characteristics when a human user conducts IEC evaluation experiments [37].

We use GMM as a pseudo-IEC user to evaluate our proposed methods. We choose four Gaussian mixture models as the basis function of mixture model. For each single model, we set *k* = 4 and dimension as 3, 5, 7, and 10. The GMM is shown in (17), and parameters *σ*  (18), *μ*  (19), and *a*_*i*_  (20) are set as follows.

### 5.3. Comparison Method: CMGA

In the experiments, we use genetic algorithm (GA) as an optimization method to evaluate the proposed methods. We compare our proposed acceleration methods with CMGA.  Algorithm 3 shows the primary process of CMGA. The difference between canonical genetic algorithm and CMGA is the judgement (steps (9)–(11)). CMGA can proceed with the next generation once average fitness of the current population is better than that of the last one.

### 5.4. Experimental Conditions

The parameter setting is in Table 2. We test with 30 trial runs of 20 generations for each GMM with different dimension setting, and apply statistical tests (sign test, Friedman test, and Bonferroni-Dunn test) to evaluate the significance of our proposals with their comparison algorithm. All these GMM tasks are posed as maximization problems with the optimal solution, which is the point with higher value.

**Figure 3:**
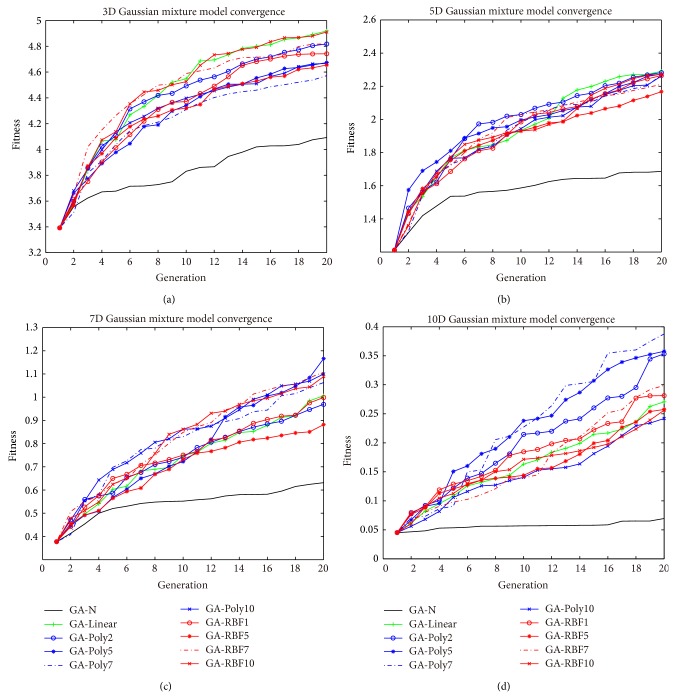
Average convergence curves of 30 trial runs with 20 generations for (a) 3D Gaussian Mixture Model, (b) 5D Gaussian mixture model, (c) 7D Gaussian mixture model, (d) 10D Gaussian mixture model, the performance of our proposed method is better than CMGA.

**Figure 4:**
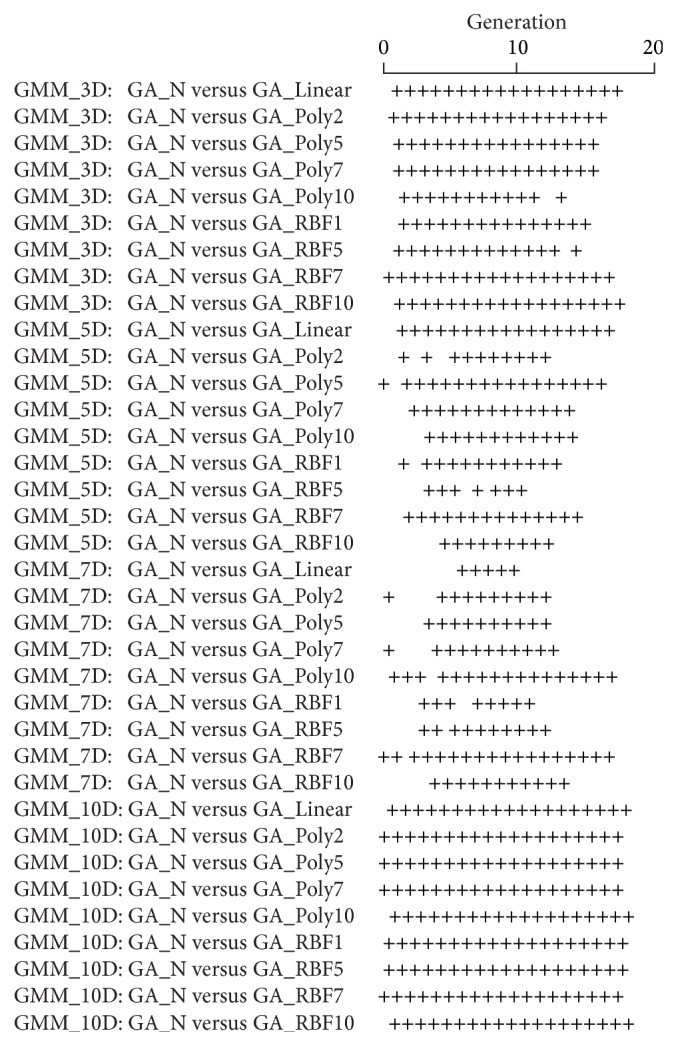
Sign test results for 30 trial runs of GA-N versus Linear, GA-N versus GA-Poly2, GA-N versus GA-Poly5, GA-N versus GA-Poly7, GA-N versus GA-Poly10, GA-N versus GA-RBF1, GA-N versus GA-RBF5, GA-N versus GA-RBF7, and GA-N versus GA-RBF10. The + mark means that a propose method converges significantly better than CMGA, respectively (*P* < 0.05). There are no cases where the proposed methods are significantly poorer than CMGA.

**Figure 5:**
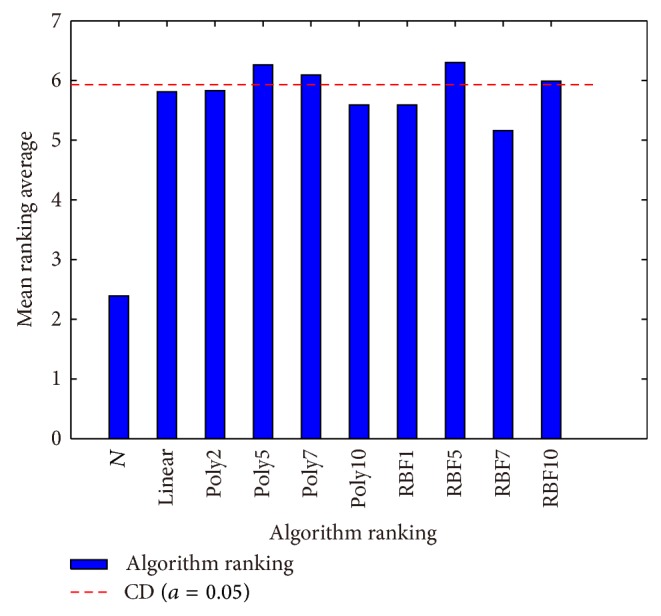
Rankings obtained through the Friedman test and graphical representation of the Bonferroni-Dunn's procedure (Taking GA-N as a control method). CD means critical difference.

We abbreviate the GA where the evolution control methods are by the linear kernel as GA-Linear, where the evolution control methods are by the polynomial kernel (15) with parameter setting with 2, 5, 7, and 10 as GA-Poly2, GA-Poly5, GA-Poly7, and GA-Poly10, where the evolution control methods are by the RBF kernel (16) with parameter setting with 1, 5, 7, and 10 as GA-RBF1, GA-RBF5, GA-RBF7, GA-RBF10, and CMGA as GA-N. These abbreviations are also used in Figures 3, 4, and 5, Tables 1 and 3.

### 5.5. Experimental Results

Figure 3 shows the average convergence curves of the best fitness values of 30 trial runs of GA-N, GA-Linear, GA-Poly2, GA-Poly5, GA-Poly7, GA-Poly10, GA-RBF1, GA-RBF5, GA-RBF7, and GA-RBF10. For different dimension GMM, Table 1 shows the mean and standard variance and Figure 4 shows their sign tests at each generation. From these results, we can obtain the following results.

1. Our proposed methods can significantly accelerate all of the GMM well.
2. The performances of nine proposed algorithms are better than that of the CMGA.
3. Linear kernel method (GA-Linear) and RBF kernel methods (GA-RBF) seem to have a better acceleration performance for the lower dimensional task (GMM with 3 dimensions); however, polynomial kernel method (GA-Poly) have a better acceleration performance for the higher dimensional task (GMM with 10 dimensions).
4. Most of the cases, RBF kernel method (GA-RBF) and polynomial kernel method (GA-Poly) have the same acceleration performance to GMM with 5 and 7 dimensions.
5. Polynomial kernel method (GA-Poly) is better than RBF kernel method (GA-RBF) in the 10-dimensional GMM.

## 6. Discussions

### 6.1. Optimization Performance of the Proposal

Experimental evaluation results show that our proposed evolution control method with human model can assist and enhance IEC search significantly. The results indicate that kernel classification is a powerful tool to establish a human model that distinguishes property of individual, which is near or beyond the global optimum (human preference). However, its performance depends on the tasks, training data, and kernel function.

From the sign test (Figure 4), we can observe that our proposed methods are more effective to the GMM with 3 and 10 dimensions than that with 5 and 7 dimensions. This result indicates that feature space projected by linear, polynomial, and RBF kernel for GMM with 3 and 10 dimensions can separate near or beyond the global optimum clearly than that for GMM with 5 and 7 dimensions. It indicates that linear, polynomial, and RBF kernel are better methods to implement human model by a linear classifier with the fitness landscape characteristics as the GMM with 3 and 10 dimensions.

From the average convergence result (Figure 3), we compare four Subfigures (a) to (d) from lower dimension to high dimension. It sketches acceleration performance of polynomial kernel seems to become better along with the GMM dimensions' increasing. It concludes that polynomial kernel methods have better performance for high dimensional tasks.

We apply Friedman test (*P* < 0.05) to rank the algorithms, which we use in the experimental evaluations (Table 3). We can observe that GA-Poly methods and GA-RBF methods almost have the same optimization performance from the ranking metric. To evaluate the significant level of all the algorithms, we apply an additional Bonferroni-Dunn test to calculate critical difference (CD in (21)) for comparing their differences in significant level of *α* < 0.05:

(21) $$\begin{array}{c} \text{CD}=q*\sqrt{\frac{k*\left(k+1\right)}{6*N}}. \end{array}$$

In (21), parameters *k* and *N* are the number of algorithms and number of benchmark tasks, respectively. They are *k* = 10 and *N* = 4 in the experimental evaluations. When *α* < 0.05, *q* is 3.261 from Table B16 (two-tailed *α*(2)) of [38].  Figure 5 sketches the results of Bonferroni-Dunn test. There is a significant difference between GA-N methods and some of our proposed methods. We can conclude that some of our proposed algorithms by embedding a human model can enhance IEC search and better than normal method (GA-N) significantly.

Our proposal is to obtain fitness landscape in feature space where the correct or accurate preference information may not be obtained in an original search space and establish a human model by these obtained information. In our proposed evolution control methods, we choose a linear classifier as a human model to obtain preference information that original search space cannot support correctly. From the experimental results, our proposed methods obtain better performance than that of the dynamic fitness threshold technique (CMGA) that conducts search strategy only based on original search space fitness landscape. In a high dimensional feature space, the important fitness landscape information is not only individuals' classification, but also the search direction or the global optimum's location, which can directly guide IEC search. If we can obtain more of such information from the designed human model in feature space, IEC performance must be improved significantly. This is the final objective of our proposal.

### 6.2. Human Model Design in Feature Space

It is crucial to design an accurate human model in feature space to obtain better performance for distinguishing individuals' property. There are three aspect issues to be considered. First is training data selection, second is kernel function selection, and the thrid is kernel parameter setting.

#### 6.2.1. Training Data

Training data selection decides the correct classification in feature space. In our experimental evaluation, we only choose three individuals with related better fitness and three individuals with related worse fitness as the labelled data for training the human model. From the result, it can be as one of the selection methods; however, if we can obtain more original search space information to decide how to select training data, it must improve human model performance by a linear classifier and reduce computational cost.

The number of training data depends on population size and linear classifier learning capability. If we separate population into two groups with better and worse fitness as the training data, the overtraining problem may happen. So how to decide the proper training data number to construct a human model is a promising topic in our future work.

#### 6.2.2. Kernel Function

Kernel function selection decides higher feature space topology. From the Mercer theorem, any kernel function corresponds to some feature spaces. It decides the individuals' distribution and classification capability of linear classifier in feature space. On one hand, in a set of well known kernel function, for a fixed search space of IEC task, there must be a kernel function with optimal classification performance. It is a promising study topic on how to select a proper kernel function for a concrete application. On the other hand, if we can obtain some a priori knowledge from an original search space, we also can design a new kernel function to transfer individuals into the desired feature space, where the fitness landscape is more beneficial for IEC search. There is not an absolute rule for selecting a proper kernel function. The design and selection of a kernel function should be adapted to a concrete search space in IEC. Some principles should be considered.

1. Kernel function selection or design should consider feature space structure, original search space's prior knowledge and training sample data.
2. Kernel function should induce a priori knowledge and present an original search space's information structure.
3. Kernel function selection and design must keep information structure in feature space, that is, linear characteristic.

#### 6.2.3. Kernel Parameter Setting

Kernel parameter setting decides the topology of a high dimensional feature space. A well-known study topic is the parameter setting and tuning, after we decide a kernel function for a concrete IEC application. There is still not a mathematical conclusion which is the best parameter setting for a kernel function. It depends on the experimental result and experience from a concrete IEC application. However, it is a valuable study topic on designing a better human model to obtain better performance in feature space for IEC application.

#### 6.2.4. Other Design Issues

In our experimental evaluation, we design a human model by a binary classifier in feature space, which is a little imprecise. The possible result is that individuals in the same generation may have the same fitness value. It decreases the pressure of selecting superior individuals in GA, which is one of drawbacks of our designed binary classifier human model. Human model design can be implemented by a multiclass classifier to solve this issue. If there is more a priori knowledge by a certain IEC human user and IEC task, we also can improve the design method of the human model to use a multi class classifier in the evolution control method. In this way, we can obtain more novel human models and evolution control methods to obtain better performance of IEC search. We will conduct this subject in our future work.

### 6.3. Computational Complexity

From (13) and Algorithm 2, it presents a concrete algorithm that conducts interactive search by using a kernel method based human model. The process needs more algebraic operations, so it is costly. For a concrete IEC application, the time used in computing is less than that of human's subjective evaluation. However, we should also consider actual time cost in kernel computing and reduce it.

In our experimental evaluation, we conduct evolution control method every generation. However, in a concrete IEC application, we can conduct this strategy several generation once to save the computational cost rather than conducting it in every generation. It is necessary to consider the time cost for this method in a real world application.

### 6.4. Proposed Methods in Other EC Algorithms and Human Related Computation

In our experimental evaluation, we apply a human model with an evolution control method in four GMM for enhancing IEC search. In general, our proposed human model can be used in all human related computing, such as awareness computing, human computing, which obtains preference information of a human user by kernel method. When applying this human model and evolution control method in another EC algorithm, we need to consider the linear classifier design method mentioned above and make sure obtained information is correct. Otherwise, the wrong information obtaining can lead to worse performance of the normal IEC search or other human related computing applications.

## 7. Conclusion and Future Work

In this study, we propose a method to relieve IEC user fatigue by establishing a human model to obtain preference information by kernel classification. In high dimensional feature space, we design a linear classifier to judge an individual property corresponding to human preference. Based on the obtained fitness landscape, we propose a human model design method and an evolution control method to enhance IEC search. The experimental evaluation with four different dimension GMM as a pseudo-IEC user shows that our proposed methods are effective. We also analyze the performance and limitation of our proposed methods. Some open topics and further opportunities are discussed.

Our further plan of this research is to evaluate our proposed methods to a concrete IEC application using a real human user to obtain a practical conclusion of the proposal. Other issues are to continue designing an efficient search strategy based on obtained human model in high dimensional fitness landscape to improve the human model by classifier design for obtaining better enhancement performance, and so forth. We will conduct these research topics in the future.

## Figures and Tables

**Algorithm 1:**
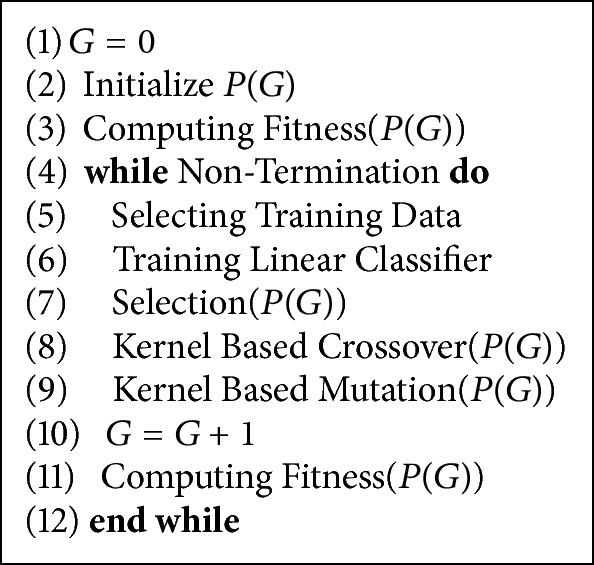
Kernel based genetic algorithm (*G*: generation and *P*(*G*): population of the *G*th generation).

**Algorithm 2:**
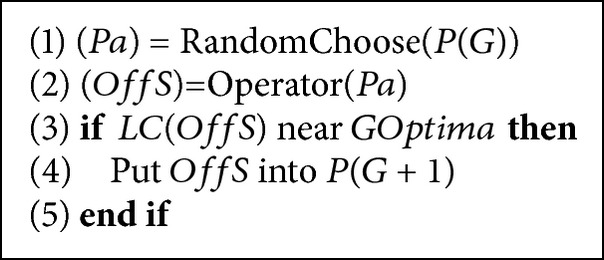
Kernel based operators (*G*: generation, *P*(*G*): population, *Pa*: parent, *OffS*: offspring, *F*(*x*): fitness function, *LC*(*x*): linear classifier, *GOptimum*: global optimum, operator: crossover or mutation).

**Algorithm 3:**
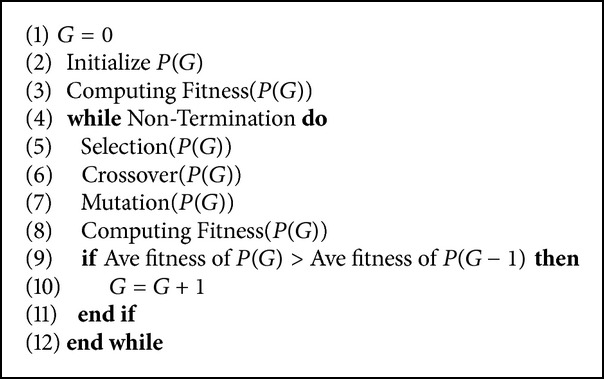
Constructive mapping genetic algorithm. (*G*: generation and *P*(*G*): population of the *G*th generation).

**Table 1:** Mean and standard variance of final results. The number in blanket is standard variance, and the bold font shows better result among the proposed methods.

*F*	*N*	Linear	Poly2	Poly5	Poly7	Poly10	RBF1	RBF5	RBF7	RBF10
3-D	4.09 (0.87)	**4.91** (**0.62**)	4.81 (0.68)	4.67 (1.10)	4.57 (0.79)	4.66 (0.91)	4.74 (0.73)	4.65 (0.76)	4.81 (0.76)	**4.91** (**0.59**)

5-D	1.68 (0.38)	**2.29** (**0.25**)	2.28 (0.32)	2.27 (0.25)	2.26 (0.32)	2.26 (0.22)	2.26 (0.31)	2.16 (0.39)	2.21 (0.35)	2.27 (0.26)

7-D	0.63 (0.20)	1.00 (0.28)	0.96 (0.30)	**1.16** (**0.38**)	1.06 (0.30)	1.09 (0.31)	0.99 (0.30)	0.88 (0.18)	1.10 (0.39)	1.08 (0.30)

10-D	0.06 (0.01)	0.27 (0.07)	0.35 (0.10)	0.35 (0.10)	**0.38** (**0.14**)	0.24 (0.04)	0.28 (0.18)	0.25 (0.06)	0.29 (0.05)	0.25 (0.04)

**Table 2:** GA parameters setting.

Parameter	Value or setting
Coding	Binary number
Number of generation	20
Population size	20
Selection	Roulette wheel and elite
	Dynamic fitness threshold
Crossover	One-point
Crossover rate	80%
Mutation rate	10%

**Table 3:** Algorithm ranking by Friedman test (*P* < 0.05).

*F*	*N*	Linear	Poly2	Poly5	Poly7	Poly10	RBF1	RBF5	RBF7	RBF10
3-D	2.53	6.22	6.03	5.40	5.52	5.15	5.63	6.42	5.65	6.45
5-D	2.87	5.97	6.00	5.97	5.93	5.57	5.83	6.13	5.07	5.67
7-D	2.67	5.80	5.13	6.83	5.87	6.33	5.67	6.27	4.40	6.03
10-D	1.50	5.27	6.13	6.83	7.03	5.30	5.23	6.37	5.53	5.80

Average	2.39	5.81	5.83	6.26	6.09	5.58	5.59	6.30	5.16	5.99

Rank	10	6	5	2	3	8	7	1	9	4
